# A decade plus of translation: what do we understand?

**DOI:** 10.1186/2001-1326-1-3

**Published:** 2012-03-30

**Authors:** Xiangdong Wang, Francesco M Marincola

**Affiliations:** 1 Department of Pulmonary Medicine, Fudan University Zhongshan Hospital, Shanghai 200032, China; 2 Biomedical Research Center, Fudan University Zhongshan Hospital, Shanghai 200032, China; 3 Infectious Disease and Immunogenetics Section (IDIS), Department of Transfusion Medicine, Clinical Center, and trans-NIH Center for Human Immunology (CHI), National Institutes of Health, Bethesda, MD 20892, USA

## Abstract

It has been more than a decade that the term "Translational" (medicine, Research, Science) has trickled through the minds of academics, clinicians, business persons, regulators, policy makers, patients and their families, patient advocates, politicians and the public. Although the term means different things for different stake holders, it reflect and eagerness to see a fruitful outcome of the resources invested in biotechnology to benefit primarily the patients but also provide financial return for those who invested. Skeptics remain who feel the concept if abused by those attempting to deviate funds for basic or clinical research to a new domain performing similar tasks under a different egida. In reality, translational sciences are not different in scope any from previous efforts to focus the goals of research toward the relevant object of helping the disabled. The difference is that, in recent decades, awareness has risen about the difficulties of reaching this goal. In particular, it has become clear that the difficulties are not limited to scientific challenges, but to a myriad of hurdles that make testing and licensing of novel concepts unnecessarily burdensome. Moreover, it was recognized that the infrastructure to support clinical research is frequently outdated and inappropriate. The biggest hurdle, however, remains the cost and the length of clinical testing that could prolong of decades the application of even the most successful treatments. As for any expanding field, a plethora of journals has appeared with "Translational" in their title. This is a positive sign of the growth in interest for the field and the need to respond to a need for editorial boards competent in the challenges of judging clinical testing. In this editorial, we will discuss the meaning of translational medicine, its goals and needs; we will summarize the remaining challenges and will provide a personal overview of the strategies that remain to be implemented.

## The public health and the health care challenge

It is expected that life expectancy will progressively increase as it has in the last decades [1]. As a consequence, the prevalence of chronic diseases, which affect predominantly the elderly, is expected to grow [2]. This will result in a formidable challenge for the health care system as chronic conditions last for a long time and require, as a consequence, prolonged spending with marginal benefit for the patient. Although the gross domestic product of a given Country is directly correlated with prolonged life expectancy [3] the relationship plateaus at the upper end of GDP values. Thus, and it is inaccurate to assume that the more it is spent in Health the better the results. A recent analysis from the United States of America Congressional Budget Office in fact suggested an inverse relationship between spending for treatment and a composite measure of quality of care [4]. Yet, the United States spending for health care continues to expand and it is projected to reach the paradoxical figure of almost 100% of the gross domestic product by the end of the this century. It could be argued that better patient selection and enhancement of treatment effectiveness may decrease the costs. Thus, it is not only in the patient interest but in the interest of the health care provider to spend a meager proportion of the astronomic health care spending in research and development relevant to these goals: this is one of the fundamental values of translational sciences.

## The need for translational sciences

It has been suggested that translational sciences are just a "fad"; a passing spree of introspection toward an utopist goal. In reality, translational sciences reflects quite specific needs that will stay relevant (independently of the term used) till they will be met. The need for translational sciences resides in 1) the need to find cost/effective solutions to the treatment of chronic diseases which in turn represent 2/3 of heath care spending in most countries; 2) the high throughput of modern biotechnology exponentially supplying novel diagnostic and therapeutic opportunities requiring efficient testing for their validation in humans; 3) the lack of accurate pre-clinical models and surrogate markers that could speed the testing of candidate products bypassing the overwhelming cost and length of clinical testing particularly for chronic diseases where long term benefit such as survival can be assessed in decades.

## The definition and goals of translational sciences

Translational sciences, also referred to as translational medicine or translational research, are interpreted differently according to the stake holder. For patients, clinicians, heath care providers, it refers to the need of accelerating the capture of the benefit of biomedical research. For the academic, translational sciences refers to the desire of confirming and validating novel concepts and identify new ones. For the commercial sector the term defines a process aimed at expediting the development and commercialization of known entities. It should be emphasized that although coming from a different prospective, the definitions are not mutually exclusive and overlap as they reflect the common need of expediting the discovery and validation process. Thus, we prefer to align the definition of translational sciences with their goals. On our account, the goals of translational sciences are to improve therapeutic outcomes by: validating the potential of novel discoveries while identifying in the process of clinical testing novel concept relevant to human disease through direct human observation. Some feel that the term "translational" was invented to redirect funds from other disciplines without providing any true conceptual advance. It is true that similar terms have been used in the past such as: pre-clinical research, clinical research, evidence-based research, disease-targeted research. And it is true that these terms reflect the same goals. Thus, translational medicine is not pretending to discover new goals and values but its essence resides more in emphasizing the need to enhance the efficiency of discovery validation recognizing the increasing obstacles. As we previously suggested, the goals are not unique to translational sciences, as basic scientists as well as physicians scientist share them; however, translational scientist, perhaps more than other, are focused and have the expertise to indentify and confront the challenges at the interface between basic and clinical investigation proposing integral solutions to increase the efficiency of the process. To quote a previous manuscript of ours: "*The traditional goals of biomedical research function as a substrate for the catalytic activity of translational research that, like an enzyme, is aimed at enhancing the efficiency rather than modifying the process*" [5].

## The challenges and potential solutions

We have extensively discussed in the past the challenges to the efficient development and validation of useful therapeutics [5-8]. We will limit the discussion the most outstanding; the first it the financial support of translational sciences. It should be emphasized that the problem is different for academia and the commercial sector. The commercial sector is not lacking funds but the problem is the containment of the cost of performing broad based clinical trials that could lead to licensing. Thus, the challenge for the commercial sector is reduction in spending. The bureaucratization of clinical research is clearly jeopardizing these efforts unnecessarily and something needs to be done to reduce the burden of clinical experimentation [5]. Better pre-clinical testing to guide the selection and predict accurately the effectiveness of therapeutics could also decrease costs. Furthermore, the identification of surrogate biomarkers that could predict the long term effectiveness of treatment (on survival) early on during a clinical trial could alleviate the costs but shortening the length of clinical investigations unlikely to be successful. These of course, are not new concepts but they remain a priority and something translational scientists should continue to focus as a priority. Yet, it is our contention that very little is done in clinical investigation to understand the mechanism of action of drugs in humans and identify causes of failure. We believe that clinical trials are still conducted sparingly with the minimal goal of testing clinical effectiveness as dictated by regulatory agencies to justify licensing but with little intent to identify causes of failure of a given treatment. In particular, little use is done of high throughput technologies that could provide mechanistic insights beyond those predicted by the trial conceptualization. We strongly believe that the bedside-to-bench direction of translational sciences remains under appreciated [8,9].

For academia the challenges are different and revolve around lack of funding; the proportions between spending to test a new concept in experimental settings are logarithmically less than those spent to validate the same further in the clinics. Few hundred thousand dollars may help a basic scientist provide a proof of concept that a new strategy is worth being investigated in the clinics; this is little money compared to the billions of dollars spend by the commercial sector to license a product. Yet, such funding is not easy to obtain. We suggest that the solution is not to compete with other academic entities such as the basic sciences but rather to identify alternative sources through public education [10] and through partnership with the commercial sector [11]. Indeed, academia can provide broader support to biotechnology development than the focused efforts of the biopharmaceutical industry and, as a consequence, play a complementary role. This applies particularly to the realm of entrepreneurship. A modern translational medicine center could provide expertise and facilities to help small biotechnology companies who cannot afford to cover all the aspects of biotechnology. Such partnership could enhance funding for academia through venture capital benefiting with its creativity the commercial sector. It should be emphasized that the proportions between spending for heath care (in the trillions) compared to the spending for research by governments (in the millions or few billions) is astounding. Yet, most of health care spending is devoted to chronic conditions for which the treatments are not effective. Wouldn't it be more logical to shift the balance toward finding new solutions rather than spending a large proportion of the gross domestic product for treatments we know do not work. As Einstein said, it is crazy to keep doing the same thing and expect different results. For health care, this is the sad reality.

Of course, there are many more challenges that translational scientists are facing, such as a fragmented infrastructure, lack of adequately trained investigators, bureaucratic and regulatory burdens, lack of support by the public which may question the value of biomedical research [12]. Yet the challenge remains in our conduct first [8]. It behooves to the translational scientists to challenge the status quo, and not to accept compromises by educating the public, patients' advocates, basic scientists, ethicists, regulatory agencies and politicians [10]. Only by changing the way translational scientists will conduct their own efforts and providing examples and proofs of principle they will be able to affect the necessary changes.

## Why a new journal of translational research

A way to chance it to provide a forum for discussion; in the last decade, several journal have emerged with the "translational" in the title reflecting this need for change. Even the American Association for the Advancement of Science has recognized this need [13]. Is there too much redundancy? Translational sciences represent a broad approach to biomedical research and do not cover specialized areas; thus, journals do not compete but rather provide broadening opportunities to disseminate ideas through editorial boards sympathetic to clinical investigation. Considering the plethora of basic science journals promoting important aspects of biomedical research but not directly relevant to public health, it is important that alternative venues are provided to clinical scientists; this is particularly important at the interface between basic and clinical investigation where the scientific output may not satisfy traditional editorial practices focus on elegance rather than usefulness. The job of the editor is critical and the ethical burden not to be underestimated. By not denying review to a manuscript, the editor assumes a responsibility that goes beyond the preference of the journal but may affect how biomedical research is rewarded and supported in the future. Paradoxically, those who passively receive the output of the scientific process become arbiters of future trends. Prestigious journal molded a field of elegance that has failed, however, to produce tangible results as most complex diseases are still uncured [14,15]. It is the task of the new journals dedicated to clinically relevant investigations to reshape the way biomedical research is perceived and rewarded. Each translational sciences journal will have the task to pursue, in unison with its peers, the fair assessment of clinical research that, perhaps less elegant and pristine than controlled laboratory investigations, will provide the much needed insights about the realities of human disease.
